# Thymoquinone Versus Metformin in Letrozole-Induced PCOS: Comparative Insights into Metabolic, Hormonal, and Ovarian Outcomes

**DOI:** 10.3390/jcm14186561

**Published:** 2025-09-18

**Authors:** Onder Ercan, Recep Dokuyucu, Ergun Yuksel, Tumay Ozgur

**Affiliations:** 1 Department of Obstetrics and Gynecology, Sular Academy Hospital, 46000 Kahramanmaraş, Turkey; 2 Department of Physiology, Medical Specialization Training Center (TUSMER), 06230 Ankara, Turkey; drecepfatih@gmail.com; 3 Department of General Surgery, Dr. Abdurrahman Yurtaslan Oncology Training and Research Hospital, University of Health Sciences, 06010 Ankara, Turkey; ergunyuksel1@saglik.edu.tr; 4 Department of Pathology, School of Medicine, Mustafa Kemal University, 31040 Hatay, Turkey; tumay.ozgur@mku.edu.tr

**Keywords:** polycystic ovary syndrome, thymoquinone, metformin, insulin resistance, lipid profile, ovarian dysfunction

## Abstract

**Objectives**: This study aimed to evaluate the effects of thymoquinone (TMQ) on metabolic, hormonal, and ovarian dysfunctions in a letrozole-induced polycystic ovary syndrome (PCOS) rat model and compare its efficacy with metformin, which is widely recognized as the first-line pharmacological treatment for PCOS. **Methods**: Thirty-two female Wistar Albino rats were randomly assigned into four groups: control (I), PCOS (II), PCOS + metformin (III), and PCOS + Thymoquinone (IV). PCOS was induced using 1 mg/kg/day letrozole for 21 days, followed by treatment with either metformin (500 mg/kg/day) or thymoquinone (50 mg/kg/day) for 30 days. Metabolic (glucose, insulin, HOMA-IR, lipid profile), hormonal (estrone, estradiol, testosterone, androstenedione), and histopathological parameters were assessed. **Results**: PCOS induction resulted in significant metabolic, hormonal, and ovarian dysfunctions. Final body weight was significantly higher in PCOS (309.0 ± 7.5 g) vs. control (275.3 ± 8.2 g, *p* < 0.001), but reduced by metformin (294.0 ± 7.4 g, *p* < 0.01) and thymoquinone (305.7 ± 7.5 g, *p* < 0.01). Glucose levels were significantly elevated in PCOS (341.8 ± 16.8 mg/dL) vs. control (260.0 ± 15.8 mg/dL, *p* < 0.01), while metformin (290.2 ± 19.7 mg/dL, *p* < 0.05) and thymoquinone (320.3 ± 13.7 mg/dL, *p* < 0.05) reduced glucose levels. Insulin and HOMA-IR were significantly increased in PCOS (*p* < 0.001), but reduced by both treatments (*p* < 0.01). Lipid profile improvements were observed, with significant reductions in TG and LDL-C and increases in HDL-C in both treatment groups (*p* < 0.05–0.01). PCOS induced hyperandrogenism, with increased testosterone and androstenedione (*p* < 0.05), and a decreased E2/E1 ratio (*p* < 0.001), which were significantly improved by metformin and thymoquinone (*p* < 0.01). Ovarian histopathology showed increased cystic and atretic follicles and reduced corpus luteum in PCOS (*p* < 0.05–0.01), which were significantly improved by both treatments. **Conclusions**: TMQ exerts metabolic, hormonal, and ovarian protective effects comparable to metformin, supporting its potential as a natural therapeutic alternative for PCOS management. Given that metformin is already established as a first-line pharmacological therapy, our findings suggest that TMQ may provide a promising complementary or alternative approach. Further clinical studies are warranted to evaluate its safety and efficacy in human PCOS patients.

## 1. Introduction

Polycystic ovary syndrome (PCOS) is one of the most prevalent endocrine disorders affecting women of reproductive age, with an estimated global prevalence of 5–15% depending on the diagnostic criteria used. It is characterized by ovarian dysfunction, hyperandrogenism, chronic anovulation, and polycystic ovarian morphology [1,2]. Beyond its reproductive manifestations, PCOS is strongly associated with insulin resistance, obesity, dyslipidemia, and an increased risk of type 2 diabetes and cardiovascular diseases [3,4]. In addition, PCOS has a profound impact on fertility, quality of life, and long-term health, further underscoring its clinical importance [3,4]. Despite its high prevalence and clinical significance, the precise etiology of PCOS remains unclear, although it is widely accepted to be a multifactorial disorder influenced by genetic, environmental, and hormonal factors.

Several experimental models have been developed to mimic the pathophysiology of PCOS in animals. Among them, the letrozole-induced PCOS model in rats has gained widespread acceptance due to its ability to replicate the key hormonal and metabolic abnormalities observed in human PCOS [5,6,7]. Letrozole, an aromatase inhibitor, suppresses the conversion of androgens to estrogens, leading to increased androgen levels, persistent anovulation, and the development of polycystic ovarian morphology [8,9]. This model closely resembles the endocrine and metabolic profile of women with PCOS, making it a valuable tool for studying potential therapeutic interventions.

Currently available treatments for PCOS include lifestyle modifications, clomiphene citrate or letrozole for ovulation induction, combined oral contraceptives, and insulin-sensitizing agents such as metformin. Among these, metformin remains the first-line pharmacological therapy due to its well-established role in improving insulin resistance, reducing hyperglycemia, and exerting anti-androgenic effects [2,10]. However, gastrointestinal intolerance and incomplete response in some patients highlight the need for additional therapeutic options.

Thymoquinone (TMQ), the main bioactive component of *Nigella sativa* (black seed), has attracted considerable attention because of its antioxidant, anti-inflammatory, anti-diabetic, and anti-apoptotic properties [10,11,12,13]. Given that oxidative stress and inflammation play key roles in the pathogenesis of PCOS [14,15], these pharmacological features provide a strong mechanistic rationale for testing TMQ in this context. By modulating insulin signaling, reducing oxidative stress, and improving hormonal balance, TMQ may mitigate the detrimental effects of PCOS on ovarian function. Importantly, while metformin represents the standard therapy, TMQ may offer a natural alternative with potentially fewer adverse effects.

Despite the growing body of evidence supporting TMQ’s benefits in metabolic and inflammatory disorders, there is a scarcity of studies systematically evaluating its efficacy in PCOS models and, in particular, in direct comparison with an established standard therapy such as metformin. Addressing this gap, our study aimed to investigate the effects of TMQ in a letrozole-induced PCOS rat model by assessing metabolic, hormonal, and histopathological parameters, with metformin as the reference comparator.

## 2. Materials and Methods

### 2.1. Study Design

A total of thirty-two female Wistar Albino rats, aged 10–12 weeks and weighing 300–350 g with normal estrous cycles, were obtained from the Mustafa Kemal University Experimental Animal Research Center (Hatay, Turkey). The number of animals was chosen in line with previous PCOS studies employing the letrozole-induced rat model [6,16], which demonstrated sufficient statistical power to detect significant differences. No formal sample size calculation was performed.

The study adhered to the principles outlined in the Guide for the Care and Use of Laboratory Animals. All experimental procedures were approved by the Animal Ethics Committee of Mustafa Kemal University (Approval number: 2014-11/9, 11 May 2014).

The rats were housed under controlled conditions (temperature 21–22 °C, humidity 55 ± 5%, 12 h light/dark cycle) with ad libitum access to food and water. A 12-day acclimatization period was provided before experimental procedures. Vaginal smears were collected once daily in the morning (08:00–10:00). Rats not showing regular estrous cycles during this phase were excluded from the study.

### 2.2. Treatment Protocol

The thirty-two rats were randomly assigned into four experimental groups (*n* = 8 each):

- Group I (control): Healthy control rats received daily oral gavage with dimethyl sulfoxide (DMSO) for 21 days.
- Group II (PCOS): Rats were induced with PCOS by oral administration of letrozole (1 mg/kg/day, dissolved in DMSO) for 21 consecutive days, followed by no further treatment.
- Group III (PCOS + MET): PCOS rats treated with metformin (500 mg/kg/day, dissolved in distilled water) for 30 days.
- Group IV (PCOS + TMQ): PCOS rats treated with thymoquinone (50 mg/kg/day, dissolved in distilled water) for 30 days.

All treatments were administered via oral gavage. Dose selection for letrozole [6,17], metformin [18], and thymoquinone [19,20,21] was based on previously published experimental studies demonstrating reproducible induction of PCOS and therapeutic efficacy in rodents.

**Figure 1:**
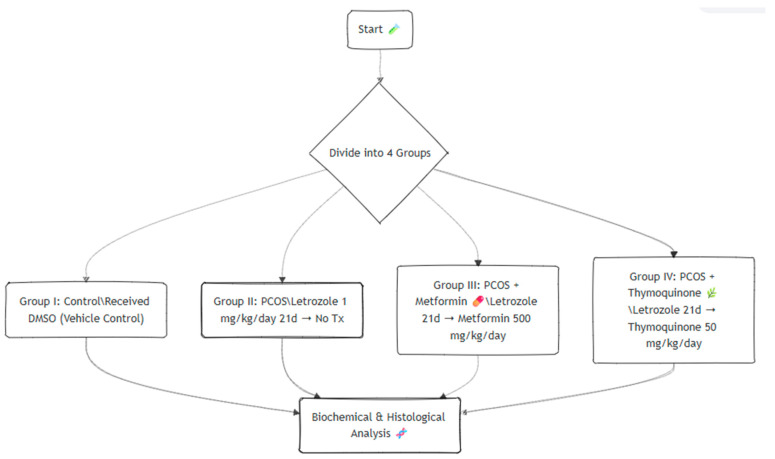
Flowchart of the study.

The development of the PCOS model was confirmed by vaginal smear cytology. Anovulation was defined as the persistence of the same predominant cell type (e.g., leukocytes or keratinized epithelial cells) for ≥10 consecutive days, consistent with established criteria [16]. At the end of the treatment period, rats were anesthetized via intraperitoneal injection of ketamine (50 mg/kg; Ketalar^®^, Abbott Laboratories, Chicago, IL, USA) and xylazine (10 mg/kg; Rompun^®^, Bayer AG, Leverkusen, Germany). Blood samples were then collected by cardiac puncture, transferred into EDTA-containing tubes, and centrifuged at 4 °C, 5000 rpm for 10 min to obtain plasma. Both ovaries were excised and fixed in 10% neutral buffered formalin for histopathological evaluation. A schematic representation of the experimental design is provided in Figure 1.

### 2.3. Histopathological Evaluation

At the beginning of the histological analysis, ovarian tissue weights were recorded. Ovaries were sectioned longitudinally along the longest axis and fixed in 10% neutral-buffered formalin for 24 h. After fixation, tissues were dehydrated through ascending ethanol series, cleared in xylene, and embedded in paraffin. Thin sections (4 µm) were prepared with a microtome and stained with hematoxylin and eosin (H&E) according to standard procedures. Slides were examined using an Olympus BX53 light microscope (Olympus, Tokyo, Japan) at 40× and 100× magnification. Follicles were classified following established histological criteria described by Amin et al. and Prabhakar et al. [16,22]. Only follicles containing an oocyte with a clearly visible nucleus were considered healthy and were categorized as the following:

➢ Primary follicles: Oocyte surrounded by a single layer of cuboidal granulosa cells, without a visible antral cavity.
➢ Preantral follicles: Multiple granulosa cell layers and a well-developed theca interna, no antral cavity.
➢ Antral follicles: Follicles with a fluid-filled antral cavity, multiple granulosa layers, and a defined zona pellucida.
➢ Atretic follicles: Follicles showing degeneration (e.g., granulosa pyknosis, oocyte shrinkage).
➢ Cystic follicles: Large fluid-filled follicles (>1 mm) with a thin granulosa layer, thickened theca interna, and no oocyte.

For quantitative analysis, the numbers of antral, atretic, and cystic follicles, as well as corpus luteum (CL) structures, were counted in each ovary. Additional morphological parameters (tunica albuginea thickness, theca interna hyperplasia, CL reduction, presence of subcapsular follicular cysts) were also assessed, particularly in control and PCOS groups.

### 2.4. Biochemical Evaluation

Serum samples were assessed for metabolic and hormonal parameters using validated biochemical techniques. Triglycerides (TG), low-density lipoprotein cholesterol (LDL-C), high-density lipoprotein cholesterol (HDL-C), and total cholesterol (Total-C) were quantified using an automated clinical chemistry analyzer (Abbott Architect C8000, Abbott Park, IL, USA). Serum insulin levels were measured via chemiluminescence immunoassay (Siemens Immulite 2000 XPI, Malvern, PA, USA). The concentrations of sex hormones, including testosterone (TT), estrone (E1), androstenedione (AS), and estradiol (E2), were determined using competitive enzyme-linked immunosorbent assay (ELISA) kits (Awareness Technology Inc., ChroMate ELISA Reader, Palm City, FL, USA). The specific ELISA product codes were E1: E-EL-0062, E2: E-EL-R0348, TT: E-EL-0072, and AS: E-EL-0068. All kits were validated for rat samples by the manufacturer, and their sensitivity and detection ranges were as follows: estrone 3 pg/mL (range 10–2000 pg/mL), estradiol 3 pg/mL (range 10–2000 pg/mL), testosterone 0.05 ng/mL (range 0.1–20 ng/mL), and androstenedione 0.05 ng/mL (range 0.1–10 ng/mL). To ensure reproducibility and accuracy, all assays were performed in duplicate.

### 2.5. Statistical Analysis

All statistical analyses were conducted using SPSS software version 26.0 (IBM Corp., Armonk, NY, USA). Data distribution was evaluated with the Shapiro–Wilk test. For normally distributed variables, comparisons among multiple groups were performed using one-way analysis of variance (ANOVA), followed by Tukey’s post hoc test for pairwise comparisons. For non-normally distributed data, the Kruskal–Wallis test was applied, with the Dunn–Bonferroni test used for post hoc analysis. Quantitative variables were expressed as mean ± standard deviation (SD) or median (interquartile range, IQR), depending on distribution. A *p*-value < 0.05 was considered statistically significant.

## 3. Results

### 3.1. Uterine and Ovarian Weights

The uterine weight was significantly lower in the PCOS group compared to the control group (*p* < 0.01). However, uterine weights significantly increased in both PCOS + MET (*p* < 0.001) and PCOS + TMQ (*p* < 0.001) groups compared to the PCOS group. Among the treatment groups, PCOS + TMQ had the highest uterine weight, indicating a more pronounced recovery effect of TMQ on uterine atrophy in PCOS-induced rats. The ovary weight was significantly higher in the PCOS group compared to the control group (*p* < 0.05), likely due to the accumulation of cystic structures. However, a significant reduction in ovary weight was observed in the PCOS + MET (*p* < 0.05) and PCOS + TMQ (*p* < 0.05) groups compared to the PCOS group, suggesting that both treatments contributed to the normalization of ovarian morphology (Table 1).

### 3.2. Histopathological Features

**Figure 2:**
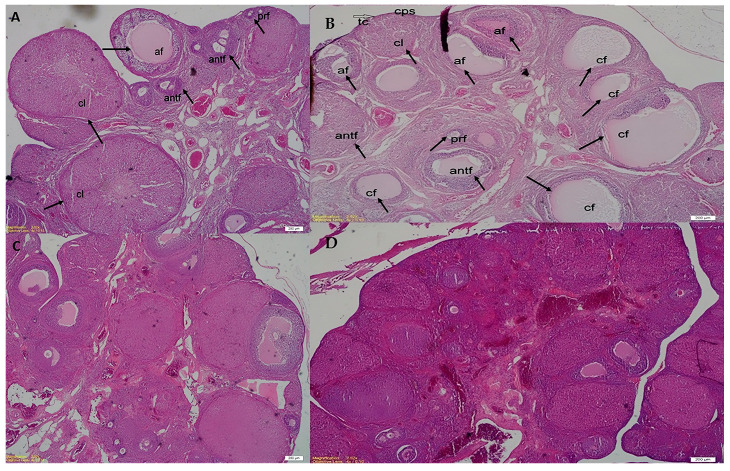
Histopathological analysis of ovarian sections in experimental groups. Representative hematoxylin and eosin (H&E, ×40) stained ovarian sections. (**A**) Control group: normal ovarian morphology with corpus luteum (cl), preantral follicles (prf), antral follicles (antf), and atretic follicles (af). (**B**) PCOS group: increased cystic follicles (cf) and atretic follicles (af), along with reduced corpus luteum (cl). (**C**) PCOS + MET group: partial restoration with decreased cystic/atretic follicles and improved corpus luteum formation. (**D**) PCOS + TMQ group: notable reduction in cystic follicles and restoration of corpus luteum, indicating improved folliculogenesis. Scale bar = 200 μm.

The PCOS group exhibited a significant reduction in preantral follicle count, although this did not reach statistical significance. A slight improvement was noted in the PCOS + MET group, while the PCOS + TMQ group showed the lowest preantral follicle count, indicating a potential remodeling effect of TMQ on follicular development. The number of antral follicles was similar across all groups, with no statistically significant differences observed (*p* > 0.05). The atretic follicle count was significantly higher in the PCOS group compared to the control (*p* < 0.05), reflecting disrupted folliculogenesis. Treatment with both metformin (*p* < 0.001) and TMQ (*p* < 0.001) significantly reduced atretic follicle numbers compared to the PCOS group, suggesting their protective roles against follicular atresia. The number of cystic follicles was markedly increased in the PCOS group compared to the control (*p* < 0.01), confirming the development of PCOS. Treatment with metformin (*p* < 0.05) and TMQ (*p* < 0.01) significantly reduced cystic follicle numbers compared to the PCOS group. Notably, the PCOS + TMQ group exhibited the lowest number of cystic follicles, suggesting a stronger effect of TMQ in reversing PCOS-associated ovarian changes. The corpus luteum (CL) count was significantly lower in the PCOS group compared to the control (*p* < 0.05), consistent with anovulatory cycles. Both treatment groups showed significant increases in CL numbers compared to the PCOS group (*p* < 0.05 for metformin and *p* < 0.01 for TMQ), indicating improved ovulatory function. In summary, TMQ treatment demonstrated a comparable, and in some cases, superior effect to metformin in restoring ovarian morphology and function, suggesting its potential therapeutic value in managing PCOS-induced ovarian dysfunction (Table 1 and Figure 2).

### 3.3. Biochemical Analysis

The biochemical parameters of the experimental groups are summarized in Table 2. The PCOS group exhibited a significant increase in final body weight compared to the control (*p* < 0.001), while both metformin and thymoquinone treatments significantly reduced body weight (*p* < 0.01). Fasting glucose levels were significantly elevated in the PCOS group (*p* < 0.01) compared to the control, and although both treatments reduced glucose levels, the difference was not statistically significant. Insulin levels and HOMA-IR were markedly higher in the PCOS group (*p* < 0.001), confirming insulin resistance, whereas metformin and thymoquinone significantly lowered these values (*p* < 0.01), with metformin showing a slightly stronger effect. Regarding lipid metabolism, triglyceride (TG) and LDL-C levels were significantly increased in the PCOS group compared to the control (*p* < 0.05), while both treatments significantly reduced these levels. Conversely, HDL-C levels were significantly lower in the PCOS group (*p* < 0.05), and treatment with both metformin and thymoquinone significantly increased HDL-C levels, with thymoquinone showing a comparable or slightly superior effect. No significant differences were observed in total cholesterol levels among the groups. These findings indicate that thymoquinone effectively improves insulin resistance and dyslipidemia in PCOS, similar to metformin (Table 2).

Hormonal parameters of the experimental groups are presented in Table 3. The PCOS group exhibited a significant increase in estrone (E1) levels compared to the control (*p* < 0.001), while both metformin and thymoquinone treatments significantly reduced E1 levels (*p* < 0.01). Similarly, estradiol (E2) levels were significantly lower in the PCOS group (*p* < 0.05) compared to the control, whereas metformin and thymoquinone treatments significantly increased E2 levels (*p* < 0.01 and *p* < 0.05, respectively), suggesting a partial restoration of estrogen balance. The E1/E2 ratio was significantly higher in the PCOS group (*p* < 0.001), indicating hormonal imbalance, while both treatments significantly reduced this ratio, with metformin showing a slightly greater effect. Testosterone (TT) levels were markedly elevated in the PCOS group (*p* < 0.05), consistent with hyperandrogenism, while both metformin and thymoquinone significantly reduced TT levels (*p* < 0.01 and *p* < 0.05, respectively). Androstenedione (AS) levels were significantly increased in the PCOS group compared to the control (*p* < 0.05), but only metformin treatment significantly lowered AS levels (*p* < 0.05). These findings indicate that both metformin and thymoquinone effectively improved hormonal imbalances in PCOS, with comparable efficacy in reducing hyperandrogenism and restoring estrogen homeostasis (Table 3).

## 4. Discussion

Our study provides novel insights into the therapeutic potential of thymoquinone (TMQ) in polycystic ovary syndrome (PCOS), representing one of the first comprehensive comparisons with metformin in a letrozole-induced rat model. We observed that TMQ improved insulin resistance, lipid profile, and ovarian morphology. Specifically, TMQ treatment lowered fasting glucose, insulin levels, and HOMA-IR, supporting its insulin-sensitizing effects. In terms of hormonal regulation, TMQ reduced testosterone and estrone while increasing estradiol, contributing to a more balanced E1/E2 ratio. Histopathological evaluation further demonstrated decreases in cystic and atretic follicles and an increase in corpus luteum counts, indicating partial improvement in folliculogenesis. These results suggest that TMQ exerts beneficial effects that in some parameters are comparable to metformin, while in others they show distinct patterns of action. Given the limited number of studies investigating TMQ in PCOS models, our findings add to the growing evidence base and may serve as a foundation for future mechanistic and clinical research.

### 4.1. Body Weight and Glucose Metabolism

Our study confirmed that PCOS induction with letrozole led to a significant increase in body weight, which was reduced by both metformin and TMQ. These findings are in line with previous reports showing that metformin decreases weight gain in PCOS models through insulin-sensitizing effects [18]. TMQ also attenuated weight gain, consistent with data by Khani et al. demonstrating that Nigella sativa supplementation reduces body weight in PCOS rats [20]. The beneficial effects of TMQ on body weight may involve modulation of glucose metabolism and inflammatory pathways, including AMPK activation [23,24].

### 4.2. Insulin Resistance

PCOS rats exhibited significant hyperglycemia, hyperinsulinemia, and elevated HOMA-IR, consistent with insulin resistance, a hallmark of PCOS [3]. Both metformin and TMQ significantly reduced insulin and HOMA-IR, although metformin showed a slightly stronger effect. These results are supported by previous studies showing that TMQ improves insulin sensitivity by reducing oxidative stress and inflammatory cytokines [23]. Malik et al. suggested that TMQ may also protect pancreatic β-cells and enhance insulin secretion [24]. The mechanisms likely involve AMPK activation and improved insulin signaling pathways such as PI3K/Akt [14].

### 4.3. Lipid Profile

Dyslipidemia was evident in PCOS rats, with increased TG and LDL-C and reduced HDL-C levels, consistent with earlier reports [25]. Both treatments improved lipid parameters; TMQ demonstrated a slightly superior effect in raising HDL-C. This effect may be attributed to its influence on lipid metabolism and PPAR-γ activation [26,27]. Our results align with findings that TMQ mitigates obesity- and diabetes-associated dyslipidemia through regulation of lipid enzymes and adipokines [26].

### 4.4. Hormonal Parameters

PCOS induction increased estrone and testosterone while reducing estradiol, leading to a higher E1/E2 ratio. Both treatments improved these parameters, although metformin was more effective in lowering androstenedione, while TMQ exerted comparable effects on estrone and testosterone. Previous studies have shown that metformin reduces hyperandrogenism by suppressing ovarian theca cell androgen production and increasing sex hormone-binding globulin (SHBG) levels [28,29], which is consistent with our finding of a stronger effect of metformin on androstenedione reduction. Yuan et al. further reported that TMQ regulates ovarian steroidogenesis by modulating CYP17A1 and CYP19A1 expression [30], which may explain its anti-androgenic effects. The partial normalization of estrogen balance by TMQ may also contribute to improved folliculogenesis.

### 4.5. Ovarian Morphology

Histopathology showed increased cystic and atretic follicles and decreased corpus luteum in PCOS rats, consistent with disrupted folliculogenesis [6,17]. Both treatments improved these features, but TMQ showed a more pronounced reduction in cystic follicles, whereas metformin was slightly superior in restoring preantral follicles. Alaee et al. demonstrated that TMQ reduces oxidative stress and apoptosis in ovarian tissue, thereby supporting follicular development and corpus luteum formation [19]. The observed morphological improvements are likely mediated by both direct antioxidant/anti-inflammatory actions and indirect hormonal regulation.

### 4.6. Clinical Translation and Mechanistic Insights

**Figure 3:**
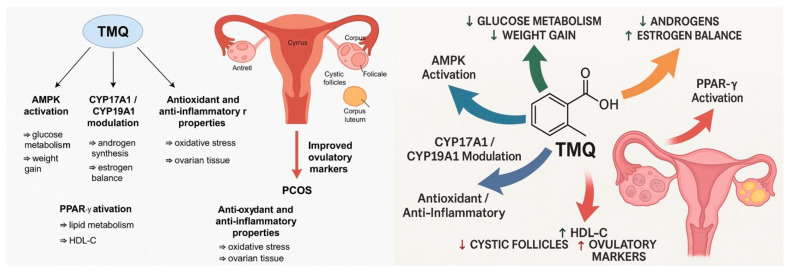
Proposed mechanisms of TMQ in PCOS. Thymoquinone exerts multiple actions: (1) AMPK activation improves glucose metabolism and reduces weight gain; (2) CYP17A1/CYP19A1 modulation decreases androgen synthesis and partially restores estrogen balance; (3) PPAR-γ activation enhances lipid metabolism and increases HDL-C; and (4) antioxidant/anti-inflammatory properties mitigate oxidative stress and protect ovarian tissue. Collectively, these effects contribute to reduced cystic follicles and improved ovulatory markers.

Our results highlight TMQ as a potential natural alternative or adjunct to metformin. To better illustrate the underlying mechanisms, we prepared a schematic summary (Figure 3) showing the principal pathways through which TMQ may act. These include AMPK activation, which improves glucose metabolism and attenuates weight gain [23,24]; CYP17A1/CYP19A1 modulation, reducing androgen synthesis and partially restoring estrogen balance [30]; PPAR-γ activation, enhancing lipid metabolism and increasing HDL-C [26]; and antioxidant and antioxidant and anti-inflammatory effects, which mitigate oxidative stress and support ovarian tissue integrity [14,15,31,32].

Although TMQ demonstrated overall effects comparable to metformin, some distinctions were observed. TMQ showed a slightly greater effect in elevating HDL-C and reducing cystic follicles, whereas metformin more strongly improved HOMA-IR and reduced androstenedione levels. These nuances underscore the need for cautious interpretation. Accordingly, we avoided overgeneralized claims such as “restores ovulation” and instead concluded that TMQ improved ovulatory markers (e.g., corpus luteum counts) rather than fully restoring ovulatory function.

Finally, we acknowledge that the letrozole-induced model may present features of relative hyperestrogenism, since aromatase inhibition can trigger compensatory increases in estrone [7,8]. This represents a limitation when extrapolating our findings to classical hyperandrogenic PCOS phenotypes.

This study has some limitations. First, although we observed significant improvements in metabolic, hormonal, and ovarian parameters, the underlying molecular mechanisms responsible for these beneficial effects were not investigated. Exploring gene and protein expression changes in key signaling pathways such as ovarian steroidogenesis, insulin signaling (AMPK, PI3K/Akt), inflammatory cascades (TNF-α, IL-6), and oxidative stress markers (Nrf2, HO-1, SOD) would greatly enhance our understanding of how thymoquinone exerts its therapeutic effects in PCOS. Furthermore, animal models, despite their usefulness in preclinical research, cannot fully replicate the complexity of human PCOS pathology. Thus, careful consideration is necessary before directly translating these findings to clinical settings. Future studies should therefore focus on identifying optimal human-equivalent dosages, evaluating pharmacokinetic properties, and confirming safety and efficacy through clinical trials. Additionally, since letrozole administration was terminated after the 21-day induction phase, spontaneous recovery of normal ovarian function and hormone levels may have started in the untreated PCOS group. This factor may have partially confounded the persistence and severity of the PCOS phenotype in that group and should be considered when interpreting between-group comparisons. Importantly, our findings that PCOS features persisted even after letrozole withdrawal are in line with recent reports that this model can produce long-lasting endocrine and morphological alterations [7,17], suggesting a semi-permanent phenotype rather than a transient state. While this strengthens the model’s translational value for chronic PCOS, it also raises questions about its reversibility compared to other models, and this must be acknowledged as a methodological limitation. Moreover, despite blinded re-evaluation of the histological slides by an independent pathologist, the possibility of minor follicle misclassification cannot be entirely excluded and remains a potential source of bias. Representative micrographs, even when annotated, may not fully capture the variability across samples, and therefore the quantitative results should be interpreted with appropriate caution. Lastly, the duration of our treatment protocol was relatively short, and long-term studies are required to determine if thymoquinone provides sustained therapeutic benefits beyond the treatment period. Despite these limitations, our study contributes valuable insights into the therapeutic potential of thymoquinone, being the first to comprehensively compare its effects with metformin in a letrozole-induced rat model of PCOS.

Additionally, our current study primarily focused on short-term metabolic, hormonal, and ovarian outcomes, and thus did not address long-term safety and comprehensive reproductive outcomes. Recent research underscores the importance of evaluating extended safety profiles, including long-term effects on fertility, endometrial health, metabolic stability, and overall quality of life in PCOS patients [2,4]. Future studies are strongly encouraged to assess these broader and long-term clinical endpoints to establish a comprehensive understanding of thymoquinone’s therapeutic potential and safety profile over extended periods.

## 5. Conclusions

In conclusion, this study demonstrated that thymoquinone effectively improves metabolic, hormonal, and ovarian dysfunctions in a letrozole-induced PCOS rat model, with effects comparable to metformin. TMQ significantly reduced insulin resistance, hyperandrogenism, and dyslipidemia while partially restoring folliculogenesis. The beneficial effects of TMQ may be attributed to its antioxidant, anti-inflammatory, and insulin-sensitizing properties, suggesting its potential as a natural therapeutic alternative for PCOS management. Given its comparable efficacy to metformin, further clinical studies are warranted to investigate the safety and effectiveness of TMQ in human PCOS patients.

## Figures and Tables

**Table 1:** Comparison of the histopathologic scores in groups (mean ± SD).

Parameters	Control (*n* = 8)	PCOS (*n* = 8)	PCOS + MET (*n* = 8)	PCOS + TMQ (*n* = 8)	*p*-Values
Uterine weight (mg)	1.0 ± 0.3	0.6 ± 0.2	3.8 ± 0.1	5.3 ± 0.2	*p* < 0.01 (control vs. PCOS); *p* < 0.001 (PCOS + MET vs. PCOS); *p* < 0.001 (PCOS + TMQ vs. PCOS)
Ovary weight (mg)	1.03 ± 0.1	1.80 ± 0.3	1.10 ± 0.1	1.37 ± 0.1	*p* < 0.05 (control vs. PCOS); *p* < 0.05 (PCOS + MET vs. PCOS); *p* < 0.05 (PCOS + TMQ vs. PCOS)
Preantral Fc	3.50 ± 0.6	2.37 ± 0.4	3.11 ± 0.6	1.66 ± 0.1	NS
Antral Fc	6.00 ± 0.7	5.25 ± 0.3	5.66 ± 0.6	6.44 ± 0.6	NS
Atretic Fc	4.62 ± 0.3	6.37 ± 0.7	2.22 ± 0.4	3.40 ± 0.3	*p* < 0.05 (control vs. PCOS); *p* < 0.001 (PCOS + MET vs. PCOS); *p* < 0.001 (PCOS + TMQ vs. PCOS)
Cystic Fc	0.5 ± 0.1	3.2 ± 0.6	0.66 ± 0.2	1.11 ± 0.4	*p* < 0.01 (control vs. PCOS); *p* < 0.05 (PCOS + MET vs. PCOS); *p* < 0.01 (PCOS + TMQ vs. PCOS)
Corpus Luteum	12.88 ± 1.2	9.12 ± 1.1	14.33 ± 1.3	15.00 ± 1.4	*p* < 0.05 (control vs. PCOS); *p* < 0.05 (PCOS + MET vs. PCOS); *p* < 0.01 (PCOS + TMQ vs. PCOS)

TMQ: thymoquinone; MET: metformin; PCOS: polycystic ovary syndrome; Fc: follicle count. Note: preantral follicles were defined as having multiple granulosa cell layers without an antral cavity. Antral follicles were defined by the presence of a fluid-filled antral cavity.

**Table 2:** Comparison of the biochemical parameters in groups (mean ± SD).

Parameters	Control (*n* = 8)	PCOS (*n* = 8)	PCOS + MET (*n* = 8)	PCOS + TMQ (*n* = 8)	*p*-Values
Initial rat weight (g)	261.6 ± 8.0	262.6 ± 6.0	269.3 ± 11.2	279.4 ± 10.7	NS
Last rat weight (g)	275.3 ± 8.2	309.0 ± 7.5 ^†^***	294.0 ± 7.4 ^†^**	305.7 ± 7.5 ^†^*	*p* < 0.001 (control vs. PCOS); *p* < 0.01 (PCOS + MET vs. PCOS); *p* < 0.01 (PCOS + TMQ vs. PCOS)
Glucose (mg/dL)	260.0 ± 15.8	341.8 ± 16.8	290.2 ± 19.7	320.3 ± 13.7	*p* < 0.01 (control vs. PCOS); *p* < 0.05 (PCOS + MET vs. PCOS); *p* < 0.05 (PCOS + TMQ vs. PCOS)
Insulin (IU/mL)	0.8 ± 0.00	3.0 ± 0.01	2.3 ± 0.01	2.6 ± 0.01	*p* < 0.001 (control vs. PCOS); *p* < 0.01 (PCOS + MET vs. PCOS); *p* < 0.05 (PCOS + TMQ vs. PCOS)
HOMA-IR	0.58 ± 0.08	2.59 ± 0.14	1.65 ± 0.15	2.05 ± 0.11	*p* < 0.001 (control vs. PCOS); *p* < 0.01 (PCOS + MET vs. PCOS); *p* < 0.01 (PCOS + TMQ vs. PCOS)
Total-C (mg/dL)	50.12 ± 2.0	56.28 ± 1.9	57.23 ± 1.7	61.07 ± 1.2	NS
TG (mg/dL)	45.53 ± 3.8	57.90 ± 2.2	50.67 ± 2.6	50.61 ± 2.3	*p* < 0.05 (control vs. PCOS); *p* < 0.05 (PCOS + MET vs. PCOS); *p* < 0.05 (PCOS + TMQ vs. PCOS)
LDL-C (mg/dL)	4.75 ± 0.3	6.62 ± 0.6	4.88 ± 0.3	4.87 ± 0.4	*p* < 0.05 (control vs. PCOS); *p* < 0.05 (PCOS + MET vs. PCOS); *p* < 0.05 (PCOS + TMQ vs. PCOS)
HDL-C (mg/dL)	14.09 ± 1.0	11.17 ± 0.4	17.44 ± 0.6	17.22 ± 1.0	*p* < 0.05 (control vs. PCOS); *p* < 0.01 (PCOS + MET vs. PCOS); *p* < 0.01 (PCOS + TMQ vs. PCOS)

TMQ: thymoquinone; MET: metformin; PCOS: polycystic ovary syndrome; HDL-C: high-density lipoprotein cholesterol; LDL-C: low-density lipoprotein cholesterol; TG: triglyceride; TC: total cholesterol; HOMA-IR: homeostasis model assessment of insulin resistance. †: initial vs. Last. * Statistically significant at *p* < 0.05; ** very significant at *p* < 0.01; *** highly significant at *p* < 0.001.

**Table 3:** Comparison of the hormone parameters in groups (mean ± SD).

Parameters	Control (*n* = 8)	PCOS (*n* = 8)	PCOS + MET (*n* = 8)	PCOS + TMQ (*n* = 8)	*p*-Values
Estrone (E1) (pg/mL)	112.7 ± 3.2	216.3 ± 2.4	190.6 ± 4.6	176.7 ± 10.3	*p* < 0.001 (control vs. PCOS); *p* < 0.01 (PCOS + MET vs. PCOS); *p* < 0.01 (PCOS + TMQ vs. PCOS)
Estradiol (E2) (pg/mL)	120.2 ± 8.6	83.2 ± 6.7	146.8 ± 15.5	122.5 ± 12.9	*p* < 0.05 (control vs. PCOS); *p* < 0.01 (PCOS + MET vs. PCOS); *p* < 0.05 (PCOS + TMQ vs. PCOS)
E1/E2 ratio	0.96 ± 0.06	2.71 ± 0.20	1.43 ± 0.18	1.53 ± 0.15	*p* < 0.001 (control vs. PCOS); *p* < 0.001 (PCOS + MET vs. PCOS); *p* < 0.001 (PCOS + TMQ vs. PCOS)
Testosterone (TT) (ng/mL)	3.55 ± 0.59	9.01 ± 0.93	4.63 ± 0.69	5.07 ± 1.15	*p* < 0.05 (control vs. PCOS); *p* < 0.01 (PCOS + MET vs. PCOS); *p* < 0.05 (PCOS + TMQ vs. PCOS)
Androstenedione (AS) (ng/mL)	0.43 ± 0.09	0.83 ± 0.11	0.64 ± 0.07	0.72 ± 0.05	*p* < 0.05 (control vs. PCOS)

## Data Availability

Data are available upon request to the corresponding author.
